# Aquatic assessment of the chelating ability of Silica-stabilized magnetite nanocomposite to lead nitrate toxicity with emphasis to their impact on hepatorenal, oxidative stress, genotoxicity, histopathological, and bioaccumulation parameters in *Oreochromis niloticus* and *Clarias gariepinus*

**DOI:** 10.1186/s12917-024-04094-9

**Published:** 2024-06-18

**Authors:** Hanan S. Khalefa, Huda O. AbuBakr, Samira H. Aljuaydi, Yousra H. Kotp, Asmaa K. Al-Mokaddem, Dalia A. Abdel-moneam

**Affiliations:** 1 Department of Veterinary Hygiene and Management, Faculty of Veterinary Medicine, Cairo University, Giza, 12211 Egypt; 2 Department of Biochemistry and Molecular Biology, Faculty of Veterinary Medicine, Cairo University, Giza, 12211 Egypt; 3 Department of Biochemistry, Faculty of Veterinary Medicine, Egyptian Chinese University, Cairo, Egypt; 4 Hydrogeochemistry Department, Desert Research Center, Cairo, 11753 Egypt; 5 Department of Pathology, Faculty of Veterinary Medicine, Cairo University, Giza, 12211 Egypt; 6 Department of Aquatic Animal Medicine and Management, Faculty of Veterinary Medicine, Cairo University, Giza, 12211 Egypt

**Keywords:** Silica-stabilized magnetite nanocomposite materials, Lead nitrate, Nile tilapia, African catfish, Hepatorenal indices, Oxidative stress, Genotoxicity, Histopathological analysis

## Abstract

**Background:**

In recent years, anthropogenic activities have released heavy metals and polluted the aquatic environment. This study investigated the ability of the silica-stabilized magnetite (Si-M) nanocomposite materials to dispose of lead nitrate (Pb(NO_3_)_2_) toxicity in Nile tilapia and African catfish.

**Results:**

Preliminary toxicity tests were conducted and determined the median lethal concentration (LC_50_) of lead nitrate (Pb(NO_3_)_2_) to Nile tilapia and African catfish to be 5 mg/l. The sublethal concentration, equivalent to 1/20 of the 96-hour LC_50_ Pb(NO_3_)_2_, was selected for our experiment. Fish of each species were divided into four duplicated groups. The first group served as the control negative group, while the second group (Pb group) was exposed to 0.25 mg/l Pb(NO_3_)_2_ (1/20 of the 96-hour LC_50_). The third group (Si-MNPs) was exposed to silica-stabilized magnetite nanoparticles at a concentration of 1 mg/l, and the fourth group (Pb + Si-MNPs) was exposed simultaneously to Pb(NO_3_)_2_ and Si-MNPs at the same concentrations as the second and third groups. Throughout the experimental period, no mortalities or abnormal clinical observations were recorded in any of the treated groups, except for melanosis and abnormal nervous behavior observed in some fish in the Pb group. After three weeks of sublethal exposure, we analyzed hepatorenal indices, oxidative stress parameters, and genotoxicity. Values of alkaline phosphatase (ALP), gamma-glutamyl transferase (GGT), urea, and creatinine were significantly higher in the Pb-intoxicated groups compared to the control and Pb + Si-MNPs groups in both fish species. Oxidative stress parameters showed a significant decrease in reduced glutathione (GSH) concentration, along with a significant increase in malondialdehyde (MDA) and protein carbonyl content (PCC) concentrations, as well as DNA fragmentation percentage in the Pb group. However, these values were nearly restored to control levels in the Pb + Si-MNPs groups. High lead accumulation was observed in the liver and gills of the Pb group, with the least accumulation in the muscles of tilapia and catfish in the Pb + Si-MNPs group. Histopathological analysis of tissue samples from Pb-exposed groups of tilapia and catfish revealed brain vacuolation, gill fusion, hyperplasia, and marked hepatocellular and renal necrosis, contrasting with Pb + Si-MNP group, which appeared to have an apparently normal tissue structure.

**Conclusions:**

Our results demonstrate that Si-MNPs are safe and effective aqueous additives in reducing the toxic effects of Pb (NO_3_)_2_ on fish tissue through the lead-chelating ability of Si-MNPs in water before being absorbed by fish.

## Introduction

Heavy metals pose a significant global hazard to living organisms due to their inherent persistence, non-biodegradability, and bioaccumulation ability [1]. Anthropogenic activities have recently released these contaminants, polluting the aquatic environment [2]. Elements like mercury (Hg), cadmium (Cd), lead (Pb), and arsenic (As) have particularly detrimental effects on ecosystems, raising serious environmental and public health concerns [3, 4]. There is a risk that they may disrupt the natural balance of aquatic and terrestrial habitats, potentially leading to the decline or extinction of certain species. Furthermore, these metals accumulate in various fish tissues and muscles, posing potential public health threats to both fish and consumers through biomagnification [5–8].

Lead represents about 0.002% of the earth’s crust, a non-disintegrable heavy metal with no nutritional value [9]. However, many studies reported neurological, reproductive, immunological, gastrointestinal, and histochemical effects caused by lead intoxication in different animal species [10–12].

Nanotechnology offers a broad range of potential applications in aquaculture, including water purification and filtration using adsorption techniques. These methods have been utilized to remove and remediate water-borne toxicity caused by heavy metal ions in aqueous media [13–15]. Additionally, nanotechnology aids in preventing and managing fish diseases, thereby enhancing fish growth, health, and productivity [16–19].

Magnetite nanoparticles (Fe_3_O_4_ NPs) are well-known iron oxide nanoparticles, playing a significant role in metal chelation due to their biochemical, catalytic, and magnetic properties [20]. Recent literature indicates that positive metal ions are electrostatically attracted to the negatively charged surfaces of Fe_3_O_4_ nanoparticles, leading to their removal from water [21, 22]. Additionally, chemical or thermal activation of these adsorbents can enhance adsorption efficiency [23].

Silicates are non-toxic, environmentally friendly, thermally and chemically stable materials with effective adsorbing power and high magnetic ability. Silica can be safely applied in the food and aquaculture sectors [24]. Combining magnetite NPs with silica has been recognized as an emerging and effective approach to improve the adsorption efficiency of these nanoparticles by enhancing their chemical stability [25, 26].

In aquatic toxicology, Nile tilapia and African catfish are extensively used as living bioindicators of water pollution due to their high sensitivity to environmental changes and ability to tolerate various stressors [27, 28]. The impact of engineered nanoparticles on aquatic organisms and the environment, including their bioavailability and potential harmful effects, can be evaluated using biological endpoints or biomarkers such as hormones, hematology, genotoxicity, biochemical parameters, and histopathology [29].

The available information on the chelating ability of silica-stabilized nanomaterials, specifically magnetite iron oxides, with lead in aquatic animals is limited [30, 31]. Therefore, this study was designed to investigate the strong magnetic properties of silica-stabilized magnetite nanocomposite materials in chelating lead. Additionally, we examined their impact on hepatorenal indices, oxidative stress biomarkers, tissue genotoxicity indicated by DNA fragmentation, residual levels of lead in fish tissues, and histopathological alterations in both Nile tilapia and African catfish.

## Materials and methods

### Synthesis of magnetite nanoparticles

The magnetite nanoparticles were prepared according to Predoi [32] using a coprecipitation technique: A solution of ferrous and ferric ion salts in water was created by adding a base at room temperature while N_2_ gas was flowing. Then, 200 ml of a 0.02 M HCl solution was added while vigorously stirring at 8000 rpm for about 30 min, following the dissolution of 4.0 ml of 1 M FeCl_3_ and 1.0 ml of 2 M FeCl_2_ in deionized, deoxygenated (DD) water. This resulted in the formation of a brown precipitate.

### Preparation of silica-stabilized magnetite (Si-M) nanocomposite materials

The (SiO_2_/Fe_3_O_4_) NPs were prepared through the hydrolysis of a silica solution obtained from rice husk [33] using the sol-gel phenomenon. This process combined 50 ml of ethanol with 0.2 g of previously prepared magnetite nanoparticles. This suspension underwent dispersion for 30 min under continuous exposure to a nitrogen flux and ultrasonication. Subsequently, 10 ml of the extracted silica solution was added, and the mixture was agitated for six hours. The resulting silica-stabilized Fe_3_O_4_ NPs (Si-MNPs) underwent multiple washes with ethanol and water before being vacuum dried for 24 h at 50^o^C to obtain the precipitate.

### Characterization of silica-stabilized magnetite (Si-M) nanocomposite materials

**Fig. 1:**
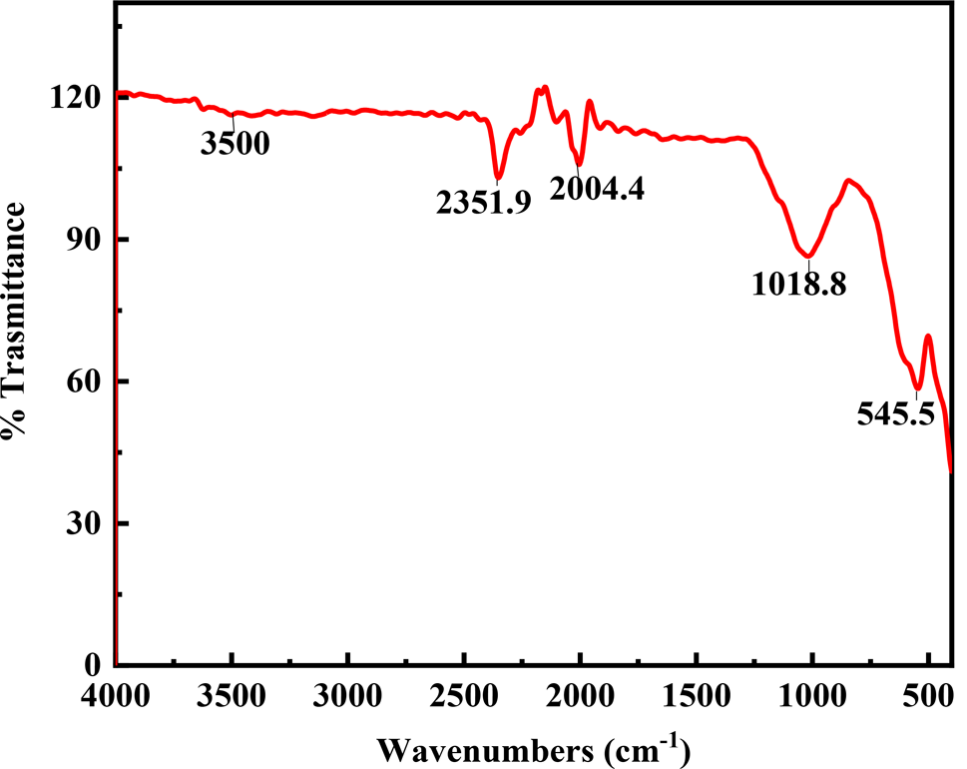
FTIR spectra of silica stabilized magnetite (Si-M) nanocomposite materials

**Fig. 2:**
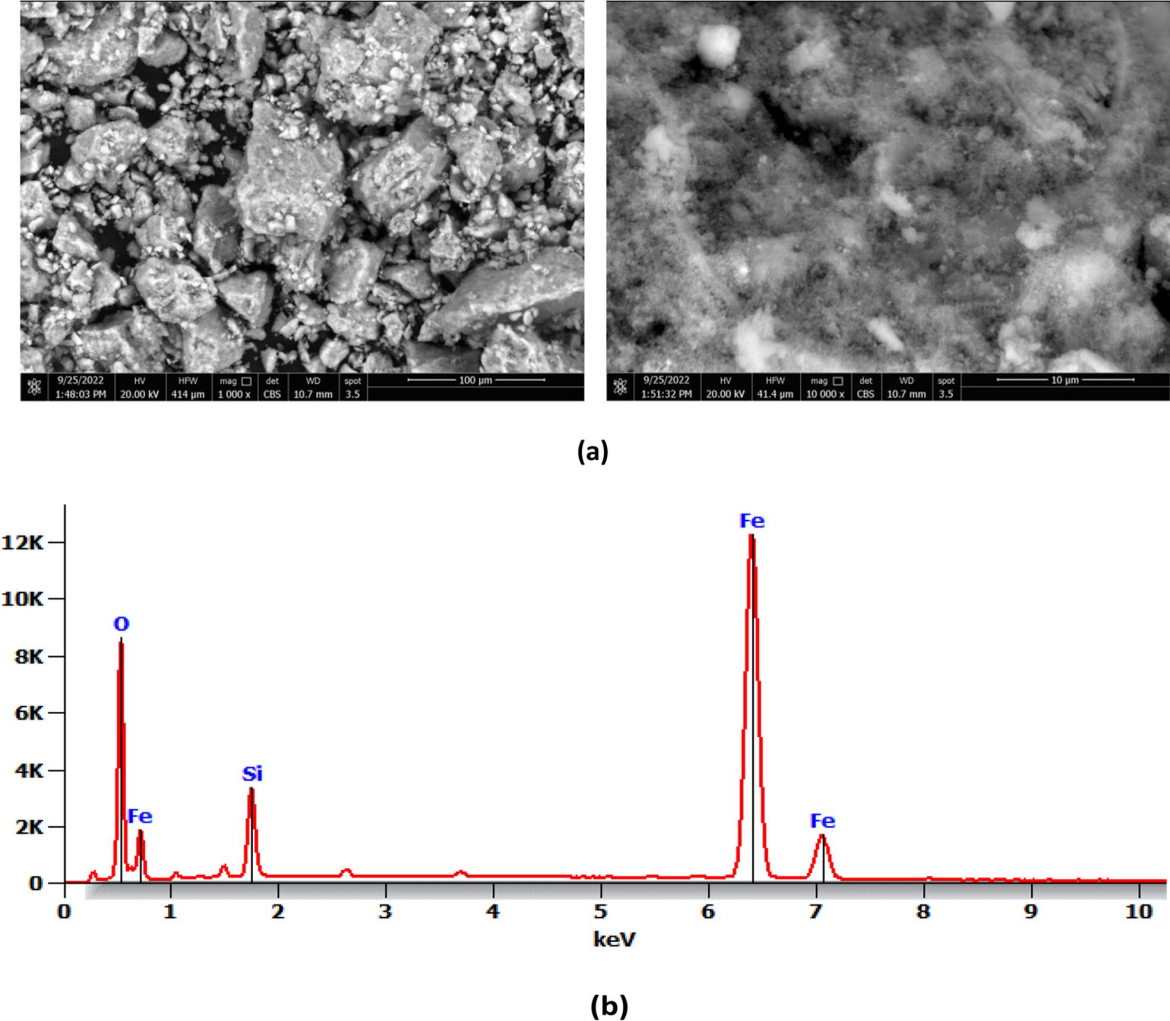
SEM images at different magnification of silica stabilized magnetite (Si-M) nanocomposite materials (**a**) and EDX analysis of silica stabilized magnetite (Si-M) nanocomposite materials

**Fig. 3:**
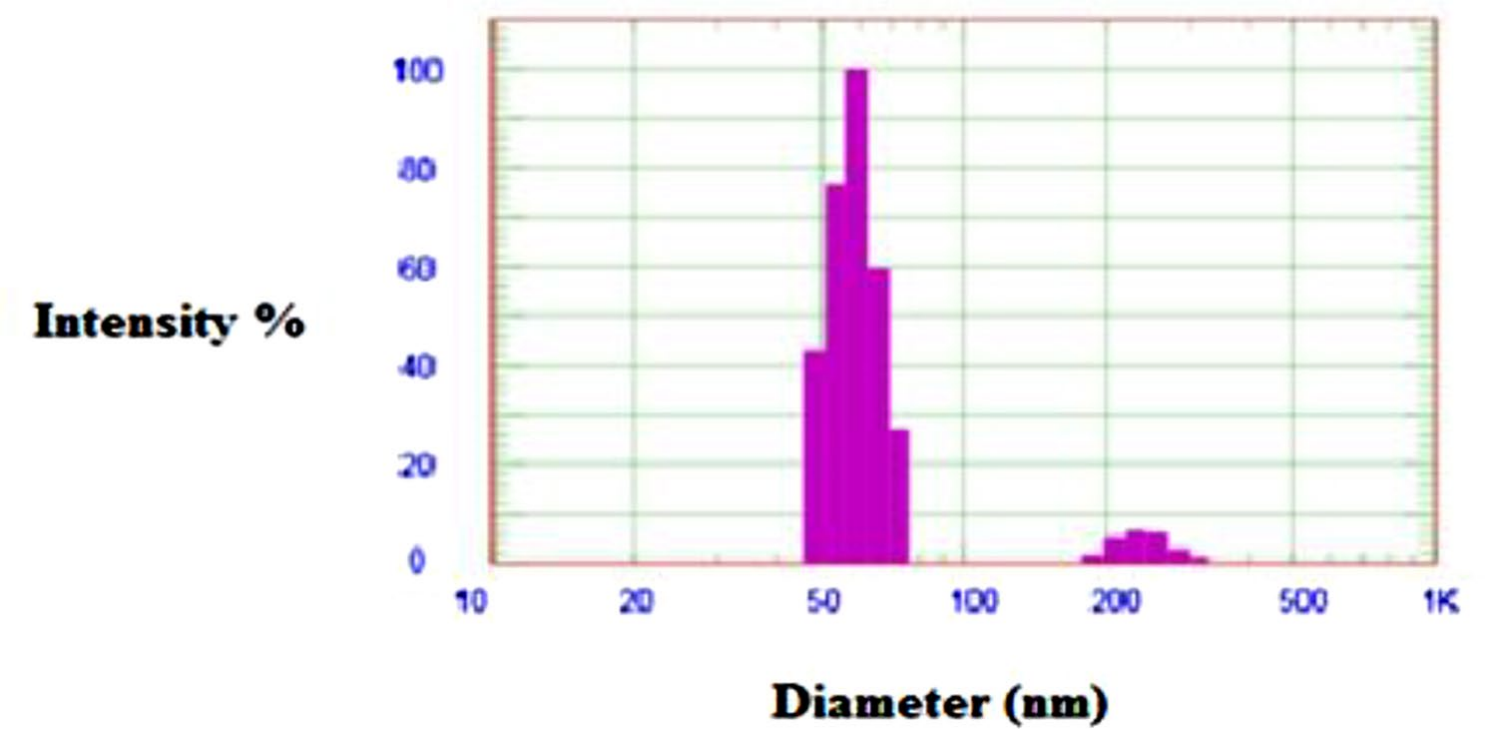
DLS of silica stabilized magnetite (Si-M) nanocomposite materials

Fourier transform infrared spectroscopy (FT-IR) of magnetite nanoparticles stabilized with silica was measured using a Nicolet Avatar 230 spectrometer with wavenumbers ranging from 400 cm^− 1^ and 4000 cm^− 1^ at a scan rate of 30 scans per minute (Fig. 1). In addition, SEM-EDAX analysis was performed for silica-stabilized magnetite nanoparticles to determine the changes in chemical constituents on their surfaces using a JEOL Quanta field emission gun (FEG) with 30 kv electrical power (Fig. 2a, b) equipped with Oxford EDAX (Japanese Corporation, Tokyo, Japan). DLS measurements using the Malvern Zeta-sized Nano-ZS nano series provide significantly better statistics than SEM; however, they necessitate more particles and commands of greater magnitude. The additional water content with the same batch of particles results in the mass distributions depicted in Fig. 3.

### Preparation of silica-stabilized magnetite (Si-M) nanocomposite materials stock solution

SiO_2_/Fe_3_O_4_ NP stock solutions were prepared by dispersing them in distilled water using a bath-type sonicator (40-kHz frequency Vibronics-250 W) over six hours, followed by 30 min of sonication each day before dosing. By using a peristaltic pump, NPs were kept suspended in water to minimize settling. At the final working concentration, the dispersion was excellent. Despite extensive sonication, aggregates of NPs were observed in the stock solution.

### Preparation of lead nitrate stock solution

The experiment utilized lead nitrate (Pb(NO_3_)_2_) from AVI-CHEM Laboratories, India. To achieve the appropriate concentrations, Pb(NO_3_)_2_ was initially dissolved in deionized water to create a stock solution (1000 ppm), which was subsequently diluted to the desired concentration before being introduced into the aquarium water.

### Experimental design

#### Lead nitrate LC_50_ value assessment

The LC_50_ treatment trial was conducted on 40 fish from each species. Nile tilapia and African catfish were divided separately into 4 groups, 10 fish in each, and exposed to 4 different concentrations of Pb(NO_3_)_2_ (3, 5, 7, and 10 mg/l) according to the study of Azua and Akaahan [34]. Fish feeding was stopped 48-h before starting the experiment to reduce basal metabolic rate and stress. Mortalities were recorded at 24, 48, 72, and 96 h. Mortality was observed and analyzed in each treatment group using the Finney probit analysis method [35].

#### Fish maintenance

Eighty apparently healthy Nile tilapia (*Oreochromis niloticus*) with an average body weight of 25 ± 5 g and 80 African catfish (*Clarias gariepinus*) with an average body weight of 60 ± 5 g were acquired from a private fish farm in Kafr El Sheikh governorate, Egypt. They were then carefully transferred to plastic fiberglass tanks equipped with aerators and transported to the wet lab of the Aquatic Animal Medicine and Management Department at the Faculty of Veterinary Medicine, Cairo University. Fish were kept for 2 weeks under observation, through which clinical examination was carried out to check the disease-free status of the fish prior to the beginning of the experiment. After acclimatization, each fish species was maintained in duplicates with a rate of 10 fish per glass aquaria (100 × 50 × 30 cm) supplied with de-chlorinated water with continuous oxygen aeration using electric air pumping compressors (Xilong, China). Fish were fed on a basal commercial diet containing 30% protein twice daily throughout the exposure duration. Water was routinely exchanged every 48 h. The physical and chemical mean values of water parameters were adjusted based on the American Public Health Association [36] guidelines as follows: dissolved oxygen, 5.5 ± 0.2 mg/l; pH 7.5 ± 0.3; temperature, 28 ± 1^o^C; unionized ammonia, 0.01 mg/l, and total alkalinity, 828 mg/l.

#### Experimental grouping

Separately, Nile tilapia and African catfish were divided into 4 groups. The first group served as the negative control group. The second group (Pb group) was exposed to 0.25 mg/l Pb(NO_3_)_2_ (1/20 of 96-h LC_50_). The third group (Si-MNPs) was exposed to silica-stabilized magnetite NPs (1 mg/l) based on the publication of Kaloyianni [37]. The fourth group (Pb + Si-MNPs) was exposed to Pb(NO_3_)_2_ simultaneously with the Si-MNPs at the same concentrations as the second and third groups. The exposure period was 3 weeks.

### Biochemical analyses of hepatorenal indices

In each group, three fish were sampled by caudal venipuncture (2 ml/fish). Following anesthetization with MS222 (50 mg/l), blood samples were collected into sterile centrifuge tubes without anticoagulant and maintained for 6 h at ambient temperature for serum separation. After centrifugation of the serum at 3000 rpm for 10 min, samples were stored at -20 °C until further use. The activity of liver function enzymes, such as alkaline phosphatase (ALP) and gamma-glutamyl transferase (GGT), were examined as described by Reitman and Frankel [38]. Kidney function indices such as urea and creatinine were measured using the Tietz method [39]. Calorimetric analysis of the biochemical tests was performed using Spectrum diagnostic kits (Spectrum Diagnostics, Cairo, Egypt) per the manufacturer’s protocol, using STAT LAB SZSL0148, version 5.

### Oxidative stress biomarkers analyses

Reduced glutathione (GSH) levels were determined according to Ellman [40]. The homogenate of liver, muscles, and gills tissues was mixed with DTNB, 0.2 M phosphate buffer (pH = 8), and 5,50-dithiobis-2-nitrobenzoic acid (DTNB). The levels of reduced glutathione are determined based on reduced DTNB levels, where glutathione produces a yellow color, and its absorbance is measured at 412 nm. Malondialdehyde (MDA) concentration was evaluated as an indicator of lipid peroxidation following the method by Ohkawa et al. [41]. The measurement involved using reactive species of thiobarbituric acid to determine MDA, with absorbance measured at 534 nm for the pink product. For protein oxidation assessment, protein carbonyl content (PCC) concentration was utilized as an index, following the procedure described by Reznick and Packer [42]. The carbonyl group was derivatized with dinitrophenylhydrazine, resulting in a stable dinitrophenylhydrazone, measured at 370 nm after derivatization.

### Assessment of tissue genotoxicity by DNA fragmentation

The DNA fragmentation was determined using the method described by Abou-Zeid et al. [43]. Briefly, hepatic, branchial, and muscular tissues weighing 10 to 20 mg each were ground in 400 ml hypotonic lysis buffers. The resulting mixture was centrifuged at 3,000 rpm for 15 min at 4 °C, and the supernatant was divided into two parts. One part was utilized for gel electrophoresis, while the other, along with the pellet, was used for measuring fragmented DNA using diphenylamine at 578 nm. The percentage of DNA fragmentation in each sample was determined using the formula: %DNA fragmentation = (OD supernatant/OD supernatant + OD pellet) × 100.

### Detection of lead residues in fish liver, gills, and muscles

After three weeks of experimental exposure, liver, gills, and muscle samples from each fish group were dissected. Approximately one gram of each organ was washed with distilled water, placed on clean slides, and dried at 70 °C for 24 h. 1 ml of concentrated HNO_3_ was added to each piece of dried tissue and placed in a clean tube for digestion. Following digestion, the samples were placed in a shaker water bath at 70 °C for four hours. Once digestion was finished, the tubes were cooled and diluted with 4 ml of distilled water, and a tissue digest aliquot was stored at room temperature. Lead detection was performed using an atomic absorption spectrophotometer (SensAA, GBC Scientific Equipment Ltd, Australia) [44, 45].

### Histopathological examination

Fish dissection was conducted, and samples of the brain, gills, liver, kidney, and spleen were collected from tilapia and catfish representing each experimental group. These specimens were preserved in neutral buffered formalin (10%) for fixation before processing in various grades of alcohols and xylenes. Subsequently, the samples were embedded in melted paraffin wax. Sections of five µm thickness were cut on glass slides and stained with hematoxylin and eosin (H&E) for light microscopy [46]. Photomicrographs were examined and captured using a Leica DM4B light microscope (Leica, Germany) and a Leica DMC 4500 digital camera (Leica, Germany).

### Statistical analysis

The significant differences among the various fish groups were analyzed using one-way ANOVA and Tukey’s multiple comparison post hoc tests using SPSS version 18. The data were presented as mean and standard error. A p-value of < 0.05 was considered statistically significant.

## Results

### Characterization of silica-stabilized magnetite (Si-M) nanocomposite materials

The FTIR results (Fig. 1) show vibrations from the Si-O-H and Si-O-Si groups. A broad band at 3500 cm^− 1^ indicates the stretching mode of the O-H group. Additionally, the SEM image (Fig. 2a) verifies the development of chemically synthesized iron oxide nanostructures, which were hexagonal and spherical. The SEM image reveals that most prepared nanoparticles were inhomogeneous, with a bright SiO_2_ spot inside a dark magnetic core-shell. Elemental composition analysis using energy dispersive X-ray spectroscopy (EDS) in Fig. 2b confirms the presence of Fe in the catalyst. Compared to SEM, DLS measurements yield better statistics due to the requirement of many particles. The DLS particle analysis confirms the presence of various nanoparticle sizes in the sample (Fig. 3).

### Lead nitrate LC_50_ short-term exposure values

No mortality was observed in the control group over the 96-hour period. The lowest concentration of lead nitrate (Pb(NO_3_)_2_) at which mortality was detected in both fish groups was 3 mg/l. The highest mortalities, with 9 Nile tilapia fish and 7 African catfish, were observed in the 10 mg/l treatment groups after 48 h (Table 1). The 96-hour LC_50_ for Nile tilapia and African catfish was recorded to be 5 mg/l. A value of 0.25 mg/l, one-twentieth (1/20) of the LC_50_ value, was used for subsequent sub-lethal studies, following the approach described by Sprague [47].

**Table 1:** Numbers of dead fish at different concentrations of Pb (NO_3_)_2_ in the 96-hour LC_50_ experiment

	Nile tilapia					African catfish				
Pb (NO_3_)_2_ (mg/l)	Control	3	5	7	10	Control	3	5	7	10
Total number of fish	10	10	10	10	10	10	10	10	10	10
Number of dead fish	0	3	5	7	9	0	2	5	6	7
Mortality percentage	0%	30%	50%	70%	90%	0%	20%	50%	60%	70%

### Clinical manifestations and mortalities of pb(NO_3_)_2_ exposed group

**Fig. 4:**
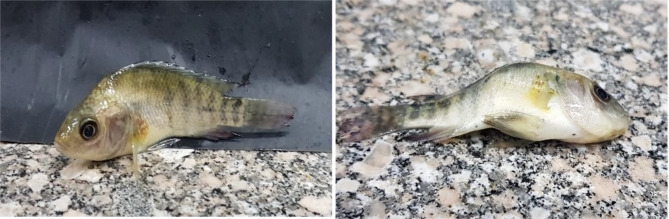
Nile tilapia in Pb exposed group showing scoliosis

During the experimental period, no mortalities were recorded in all groups. However, the Nile tilapia fish group exposed to lead nitrate in water showed melanosis, abnormal nervous and swimming behavior, abnormal rapid movement of pectoral fins, and skeletal deformities such as scoliosis (Fig. 4).

### Hepatorenal indices analyses

Liver function enzymes, including alkaline phosphatase (ALP) and gamma-glutamyl transferase (GGT), were measured in the serum of Nile tilapia and African catfish from all experimental groups. The levels of ALP and GGT in both tilapia and catfish showed a significant increase in the Pb group compared to the Si-MNPs, Pb + Si-MNPs, and control groups. Additionally, kidney function tests for urea and creatinine measured in tilapia fish serum across all groups demonstrated significantly higher levels in the Pb group compared to the other groups. In contrast, there was no marked significance in catfish serum creatinine levels between the Pb-exposed and other groups (Table 2).

**Table 2:** Hepatorenal function indices measured in Nile tilapia and African catfish

Fish Groups	Nile tilapia serum				African catfish serum			
	ALP (U/L)	GGT (U/L)	Urea (mg/dl)	Creatinine (mg/dl)	ALP (U/L)	GGT(U/L)	Urea(mg/dl)	Creatinine(mg/dl)
Control	160.23 ± 5.19^b*^	137.62 ± 7.00^b^	0.87 ± .28^b^	0.52 ± 0.03^b^	132.41 ± 20.50^b^	2.71 ± 0.029^b^	13.54 ± 0.14^b^	0.23 ± 0.01
Pb group	211.13 ± 16.40^a^	268.02 ± 57.64^a^	6.58 ± 1.84 ^a^	0.73 ± .03^a^	190.89 ± 3.15^a^	6.33 ± 1.17^a^	24.33 ± 2.33^a^	0.41 ± 0.044
Si-MNPs group	167.64 ± 1.65^b^	177.30 ± 4.79^ab^	1.57 ± 0.46^b^	0.59 ± .02^b^	162.92 ± 3.08^ab^	4.42 ± 0.21^ab^	15.44 ± 1.02^b^	0.24 ± 0.07
Pb + Si-MNPs group	169.04 ± 4.78^b^	225.18 ± 53.61^a^	3.71 ± 0.90 ^ab^	0.62 ± .030^ab^	171.20 ± 1.69^ab^	6.33 ± 0.35 ^a^	17.54 ± 0.35^b^	0.27 ± 0.01
*P* value	0.015	0.016	0.020	0.002	0.026	0.009	0.002	0.062

* ^a, b^ different letters in the same columns refer to statistical significance between groups (*p* value ≤ 0.05). Pb Group: lead nitrate Pb(NO_3_)_2_ exposed group; Si-MNPs group: silica-stabilized magnetite (Si-M) NPs exposed group; Pb + Si-MNPs group: exposed simultaneously to lead nitrate and silica-stabilized magnetite (Si-MNPs)

### Oxidative stress biomarkers

#### Reduced glutathione findings

In Nile tilapia, the concentration of GSH significantly decreased in the Pb group to 0.3, 0.2, and 0.25 in the liver, gills, and muscles, respectively, compared to the control group. However, its concentration returned nearly to normal levels in the liver and gills of the Pb + Si-MNPs group. Interestingly, the Pb + Si-MNPs group showed a significant increase in GSH concentration in the muscles compared to the control group (Table 3).

**Table 3:** GSH concentrations in Nile tilapia and African catfish

Fish Groups	Nile tilapia			African catfish		
	Liver	Gills	Muscle	Liver	Gills	Muscle
Control	1.2 ± 0.2^b^*	1.1 ± 0.2^b^	1.1 ± 0.2^b^	5.13 ± 0.2^d^	2.5 ± 0.3^c^	2.1 ± 0.2^b^
Pb group	0.3 ± 0.1^a*^	0.2 ± 0.05^a^	0.25 ± 0.03^a^	1.03 ± 0.3^a^	0.43 ± 0.1^a^	0.4 ± 0.1^a^
Si-MNPs group	2.7 ± 0.1^c^*	2.1 ± 0.4^c^	1.2 ± 0.1^b^	2.07 ± 0.2^b^	1.23 ± 0.1^b^	1.73 ± 0.2^b^
Pb + Si-MNPs group	0.9 ± 0.06^b^	1.1 ± 0.2^b^	3.6 ± 0.1^c^	3.17 ± 0.2^c^	2.13 ± 0.2^c^	2.77 ± 0.1^c^

* ^a, b,c,^ different letters in the same columns refer to statistical significance between groups (*p* ≤ 0.05). Values are means ± SE, *n* = 5. GSH: Glutathione reduced (nM/mg protein). Pb Group: lead nitrate Pb(NO_3_)_2_ exposed group; Si-MNPs group: silica-stabilized magnetite (Si-M) NPs exposed group; Pb + Si-MNPs group: exposed simultaneously to lead nitrate and silica-stabilized magnetite (Si-MNPs)

In African catfish, the concentration of GSH significantly decreased in the Pb intoxicated group from 5.13, 2.5, and 2.1 to 1.03, 0.43, and 0.4 in the liver, gills, and muscles, respectively, compared to the control group. In contrast, its concentration was significantly elevated in the Pb + Si-MNPs group to 3.17, 2.13, and 2.77, respectively.

#### Lipid peroxidation findings

In Nile tilapia, MDA concentration significantly increased in the Pb group to 44.7, 31.7, and 12.6 in the liver, gills, and muscles, respectively. However, in the Pb + Si-MNPs group, MDA concentration significantly decreased in the liver and gills to 18 and 13, respectively, and returned nearly to levels comparable to the control group (Table 4).

**Table 4:** MDA concentrations in Nile tilapia and African catfish

Fish Groups	Nile tilapia			African catfish		
	Liver	Gills	Muscle	Liver	Gills	Muscle
Control	7.8 ± 0.4^a*^	6 ± 0.6^a^	5.5 ± 0.3^a^	11 ± 0.6^a^	6 ± 0.6^a^	1.77 ± 0.1^a^
Pb group	44.7 ± 4.3^c^	31.7 ± 0.9^d^	12.6 ± 0.9^b^	40.67 ± 2.3^c^	26 ± 2^c^	14.67 ± 1.4^c^
Si-MNPs group	11.5 ± 0.3^ab^	9.2 ± 0.6^b^	6.1 ± 0.2 ^a^	22.67 ± 1.5^b^	15 ± 1.2^b^	8.5 ± 0.3^b^
Pb + Si-MNPs group	18 ± 1.2^b^	13 ± 0.6^c^	7 ± 0.6 ^a^	15.67 ± 0.9^a^	8.5 ± 0.3^a^	3 ± 0.5^a^

* ^a, b,c, d^ different letters in the same columns refer to statistical significance between groups (*p* ≤ 0.05). Values are means ± SE, *n* = 5. MDA: Malondialdihyde (nM/mg protein). Pb Group: lead nitrate Pb(NO_3_)_2_ exposed group; Si-MNPs group: silica-stabilized magnetite (Si-M) NPs exposed group; Pb + Si-MNPs group: exposed simultaneously to lead nitrate and silica-stabilized magnetite (Si-MNPs)

In African catfish, the concentration of MDA significantly increased to 40.67, 26, and 14.67 in the liver, gills, and muscles, respectively, in the Pb group compared to the control group. However, its concentration significantly improved and nearly reached control levels in the Pb + Si-MNPs group, measuring 15.67, 8.5, and 3, respectively.

#### **Protein oxidation findings**

In Nile tilapia, the PCC concentration significantly increased to 13, 15, and 11 in the Pb group’s liver, gills, and muscles, respectively, compared to the control group. However, its concentration was mitigated, nearly returning to control levels in the Pb + Si-MNPs group (Table 5).

**Table 5:** Protein oxidation concentrations in Nile tilapia and African catfish

Fish Groups	Nile tilapia			African catfish		
	Liver	Gills	Muscle	Liver	Gills	Muscle
Control	2 ± 0.2^a*^	1.6 ± 0.1^a^	1.5 ± 0.1^a^	2.07 ± 0.3^a^	1.5 ± 0.3^a^	1.83 ± 0.2^a^
Pb group	13 ± 0.5^c^	15 ± 1^c^	11 ± 1^c^	14 ± 0.6^c^	17 ± 0.6^c^	8 ± 0.6^c^
Si-MNPs group	7 ± 0.7^b^	7.5 ± 0.5^b^	6.5 ± 0.5^b^	9 ± 0.6^b^	8.5 ± 0.3^b^	5.17 ± 0.4^b^
Pb + Si-MNPs group	3 ± 0.3^a^	2 ± 0.05^a^	2 ± 0.05^a^	3.5 ± 0.3^a^	2.5 ± 0.3^a^	2.83 ± 0.4^a^

* ^a, b,c,^ different letters in the same columns refer to statistical significance between groups (*p* ≤ 0.05). Values are means ± SE, *n* = 5. PCC: Protien carbonyl content (nM/gm protein). Pb Group: lead nitrate Pb(NO_3_)_2_ exposed group; Si-MNPs group: silica-stabilized magnetite (Si-M) NPs exposed group; Pb + Si-MNPs group: exposed simultaneously to lead nitrate and silica-stabilized magnetite (Si-MNPs)

Similarly, in African catfish, the concentration of PCC significantly increased to 14, 17, and 8 in the liver, gills, and muscles, respectively, in the Pb group compared to the control group. However, its concentration significantly improved and nearly reached control levels in the Pb + Si-MNPs group, measuring 3.5, 2.5, and 2.83, respectively.

### Tissue genotoxicity and DNA fragmentation findings

In Nile tilapia, the percentage of DNA fragmentation significantly increased in the Pb group to 79.3, 58, and 66 in the liver, gills, and muscles, respectively, compared to the control group. Conversely, its percentage significantly decreased in the liver, gills, and muscle tissues in the Pb + Si-MNPs group (Table 6).

**Table 6:** DNA fragmentation percentage in Nile tilapia and African catfish

Fish Groups	Nile tilapia			African catfish		
	Liver	Gills	Muscle	Liver	Gills	Muscle
Control	9 ± 0.6^a*^	7.2 ± 0.4^a^	11 ± 0.6^a^	8.5 ± 0.3^a^	7 ± 0.5^a^	11.5 ± 0.2^a^
Pb group	79.3 ± 5.2^c^	58 ± 4.6^c^	66 ± 6.4^c^	66.6 ± 0.6^c^	50 ± 2^c^	59 ± 0.6^c^
Si-MNPs group	11.7 ± 0.9^ab^	14 ± 0.6^ab^	13.3 ± 0.9^a^	11 ± 0.2^asb^	13 ± 0.1^ab^	13 ± 0.4^a^
Pb + Si-MNPs group	20 ± 1.2^b^	16.7 ± 0.9^b^	25 ± 2.9^b^	25 ± 0.5^b^	15.6 ± 0.8^b^	23 ± 0.4^b^

* ^a, b,c,^ different letters in the same columns refer to statistical significance between groups (*p* ≤ 0.05). Values are means ± SE, *n* = 5. PCC: Protien carbonyl content (nM/gm protein). Pb Group: lead nitrate Pb(NO_3_)_2_ exposed group; Si-MNPs group: silica-stabilized magnetite (Si-M) NPs exposed group; Pb + Si-MNPs group: exposed simultaneously to lead nitrate and silica-stabilized magnetite (Si-MNPs)

Similarly, in African catfish, the percentage of DNA fragmentation significantly increased in the Pb-intoxicated group to 66.6, 50, and 59 in the liver, gills, and muscles, respectively, compared to the control group. However, its percentage significantly decreased in all tissues in the Pb + Si-MNPs group to 20, 16.7, and 25, respectively, compared to the Pb group.

### Lead residues accumulation in the liver, gills, and muscles

**Fig. 5:**
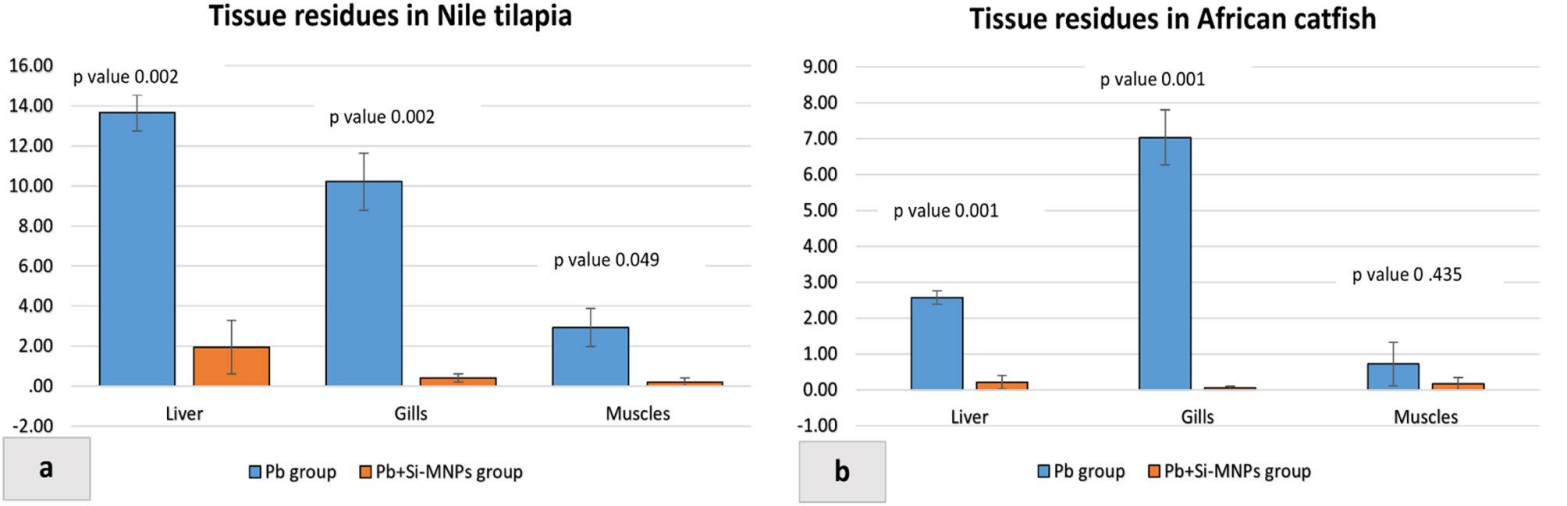
(**a, b**) Lead residues in liver, gills and muscles of experimental Nile tilapia and African catfish

The accumulation of Pb was measured and recorded in the liver, gills, and muscles of Nile tilapia and African catfish. Pb residues in the liver, gills, and muscles of Nile tilapia and African catfish were significantly higher in the Pb-exposed group compared to the Pb + Si-MNPs group (Fig. 5a, b).

In Nile tilapia, high Pb accumulation was observed in the liver, followed by the gills, while in African catfish, high Pb accumulation was observed in the gills, followed by the liver. The lowest Pb concentration was recorded in the muscles of both species.

### Histopathological findings

**Fig. 6:**
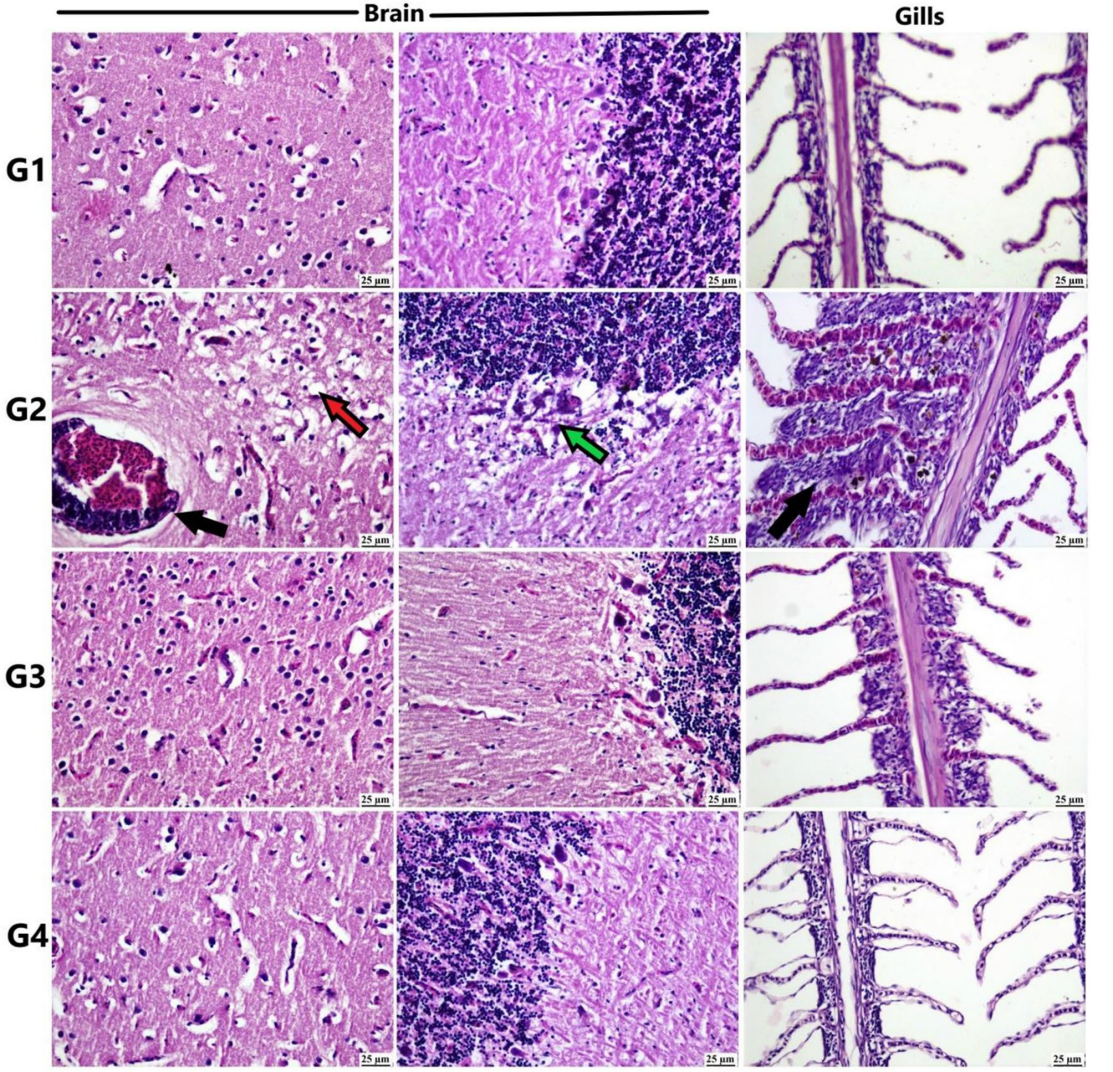
Photomicrographs of different organs of Nile tilapia (H&E). Control group showing normal histological structure of all examined organs, Pb group: vacuolation (red arrow) and perivascular inflammatory cells infiltration (black arrow) and focal gliosis (green arrow) in brain. Gill hyperplasia and fusion (black arrow). Si-MNPs group: showing apparently normal structure of brain and gills Pb + Si-MNPs group showing apparently normal brain and gills

**Fig. 7:**
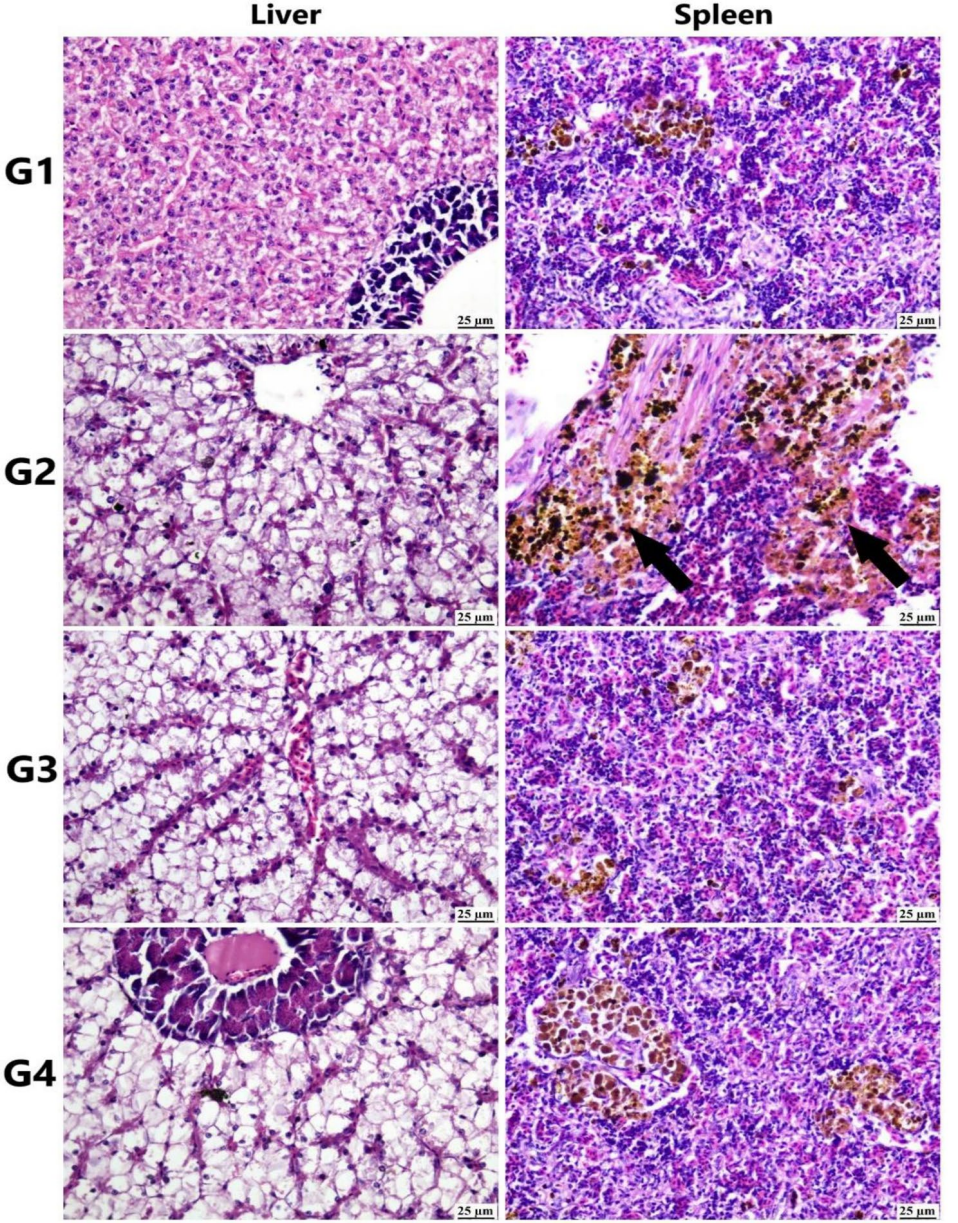
Photomicrographs of different organs of Nile tilapia (H&E). Control group showing normal histological structure of all examined organs, Pb group showing Marked liver vacuolation. Hyperplasia in melano-macrophage centers (black arrows) in spleen. Si-MNPs group showing hepatocellular vacuolation in liver and apparently normal spleen. Pb + Si-MNPs showing hepatocellular vacuolation and mild melano-macrophage center activation in spleen

The histopathological evaluation of the collected tissue samples from Nile tilapia is depicted in Figs. 6 and 7. In the brain of tilapia fish from the Pb group, marked vacuolation, vasculitis, perivascular mononuclear inflammatory cell infiltration, and gliosis were observed. Conversely, other groups exhibited apparently normal brain structure.

The gills from the control and Si-MNPs groups showed histologically normal features, while the Pb group exhibited gill hyperplasia and fusion of secondary gill lamellae with intense inflammatory cell infiltration. The Pb + Si-MNPs group showed apparently normal gills.

In the liver sections, except for the control group, the rest of the experimental groups exhibited hepatocellular vacuolation. Hyperplasia and activation of melano-macrophage centers were detected in spleen specimens from the Pb groups, while apparently normal spleen was observed in the other experimental groups.

**Fig. 8:**
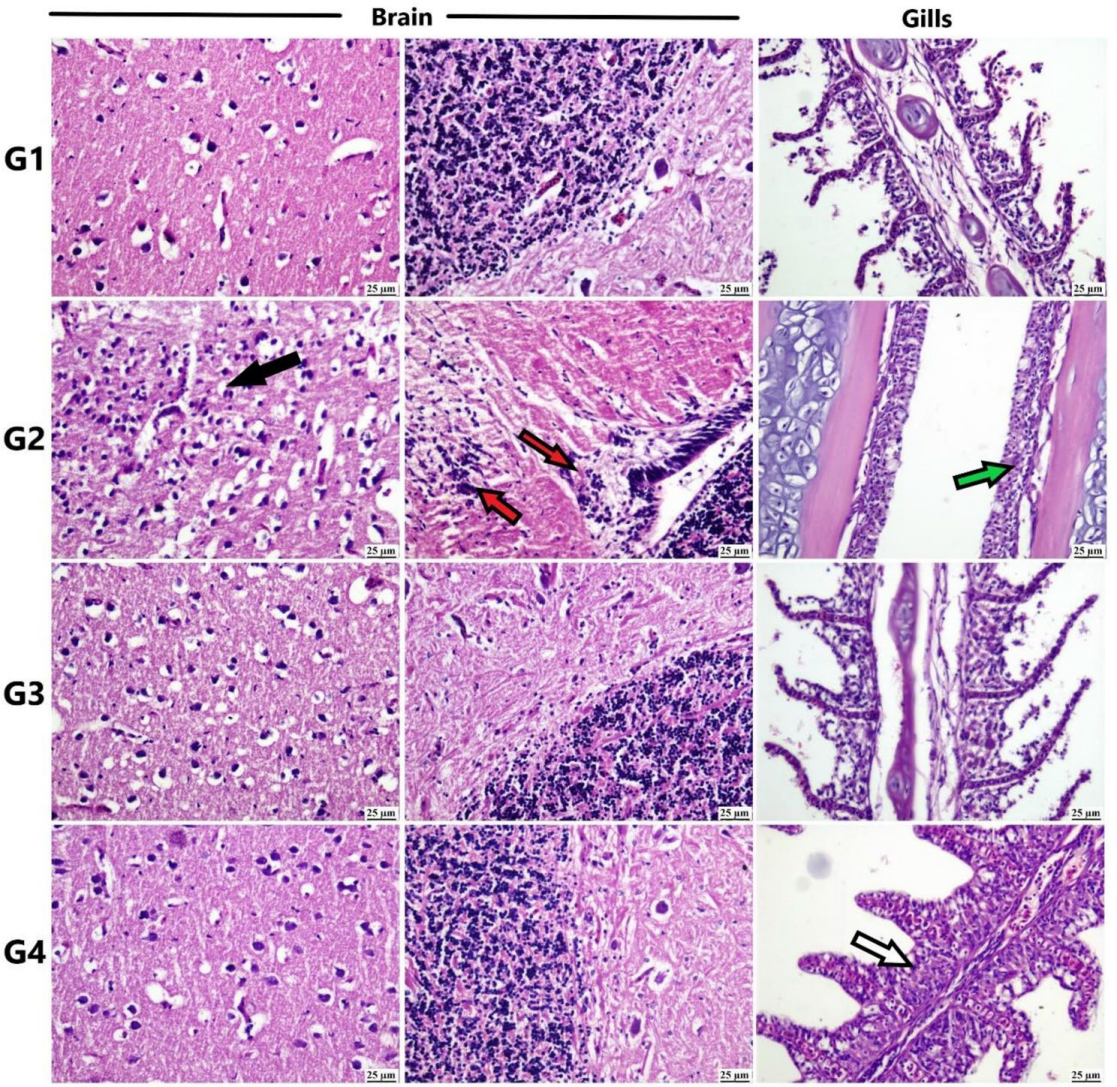
Photomicrograph of different organs from African catfish (H&E). Control group showing normal histological structure of different organs. Pb group showing neuronal degeneration (black arrow) and focal gliosis (red arrow) in brain, destruction of secondary gill lamellae (green arrow). Si-MNPs group showing apparently normal structure of different organs. Pb + Si-MNPs group showing apparently normal brain and inflammatory cells infiltration in gills (white arrow)

**Fig. 9:**
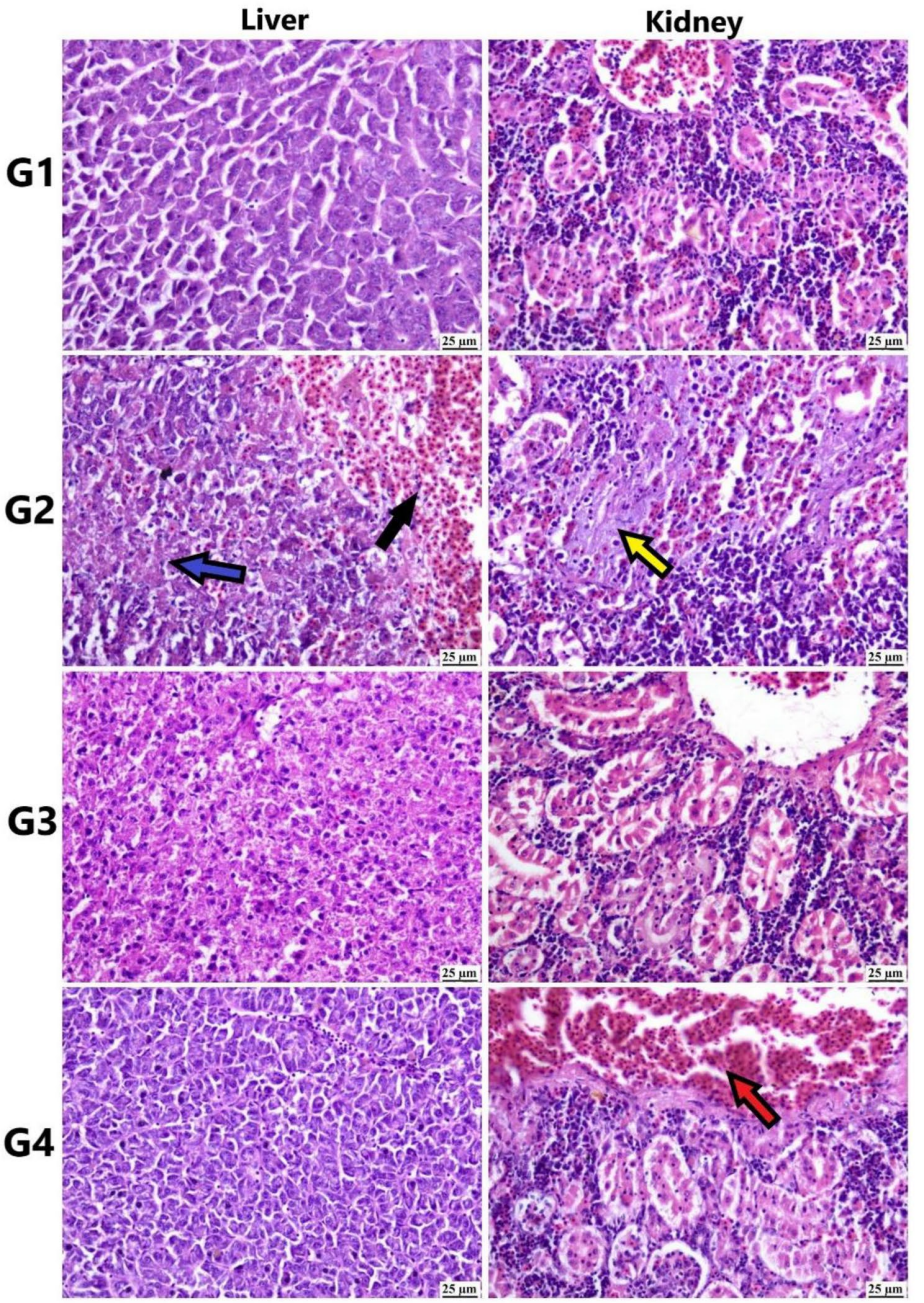
Photomicrograph of different organs from African catfish (H&E). Control group showing normal histological structure of different organs. Pb group showing hepatocellular necrosis (blue arrow) and hemorrhage (black arrow) in liver. Necrosis in renal tubules (yellow arrow) of kidneys. Si-MNPs group showing apparently normal structure of different organs. Pb + Si-MNPs showing apparently normal liver and congestion (red arrow) in kidneys

Microscopic examination of the different tissue samples from African catfish (Figs. 8 and 9) revealed an absence of detectable histopathological changes in the control and Si-MNPs groups. In the Pb groups, focal gliosis, edema, and neuronal degeneration were observed in brain sections. The Pb + Si-MNPs groups showed mild neuronal edema in a few sections, while most examined sections appeared normal.

Destruction of the secondary gill lamellae and mononuclear inflammatory cell infiltrations were observed in the Pb groups. Additionally, inflammation was noted in the gill tissue from the Pb + Si-MNPs groups.

Hepatocellular necrosis and hemorrhage were frequently detected in the liver of the Pb group, while the Si-MNPs groups exhibited apparently normal liver tissue. Renal tubular damage was subsided in kidney sections from the Pb + Si-MNPs groups, with mild vascular congestion observed in a few individuals.

## Discussion

Aquatic organisms, particularly fish, are primary targets for heavy metal contamination and accumulation, making them valuable indicators of aquatic pollution levels [48]. In aquaculture, innovative strategies such as feed additives, probiotics, and nanoparticles offer cost-effective and environmentally friendly methods to control heavy metal contamination [49]. Metallic nanoparticles, including silica-stabilized magnetite (Si-M) nanocomposite materials, have gained widespread use in various applications like biomedical, food, agricultural, and electronics sectors due to their unique physical and chemical properties [50].

The superparamagnetic properties of silicates and magnetite (Fe_3_O_4_) nanoparticles make silica-stabilized magnetite (Si-M) nanocomposite materials effective in pharmaceutical and agricultural applications and the separation of heavy metals and toxic ions from water [51].

The characterization of the silica-stabilized magnetite nanocomposites used in the study is depicted in Fig. 1, demonstrating the presence of silica networks on the magnetite surface through Fe-O-Si bonds as indicated by significant FT-IR spectra [52]. Notably, a strong absorbance band at 1018 cm^− 1^ in Fig. 1 is attributed to the vibration of the Si-O-H and Si-O-Si bonds. Additionally, weak absorption peaks observed are attributed to vibrations of the C-H bonds of organic groups that remained during preparation [53]. The broadband observed at 3500 cm^− 1^ indicates the stretching mode of the O-H group, representing the water content of the silica-stabilized magnetite nanocomposite samples [54]. The distinctive Fe_3_O_4_ diffraction peaks are somewhat weakened due to the silica coat and mixed groups, allowing for the detection of amorphous silica diffraction peaks [55, 56].

Furthermore, Fig. 2a presents a typical FE-SEM image of magnetite nanoparticles stabilized with silica. The SEM image reveals that most nanoparticles are non-homogeneous, with a bright SiO_2_ spot inside the dark magnetic core-shell structure. An energy-dispersive X-ray spectroscopy (EDS) analysis was conducted on a silica-stabilized magnetite nanocomposite sample.

Figure 2b displays the EDS results derived from SEM analysis of the nanomaterial mentioned above, affirming the presence of Fe in the silica-stabilized magnetite (Si-M) NPs. Furthermore, the presence of O, Si, and Fe, along with the Fe peak exhibiting higher intensity than the Si peak, suggests the successful preparation of silica-stabilized magnetite nanocomposite materials.

Compared to SEM, DLS measurements offer significantly improved statistical data as they involve a larger number of particles. The DLS particle analysis further confirms the presence of various nanoparticle sizes within the sample, as illustrated in Fig. 3.

The silica-stabilized magnetite NPs were recently used in the aquaculture industry. In our study, 1 mg/l of Si-MNPs were added to the aquaria water. Based on studies by Zhu et al. [30], Kaloyianni et al. [37], and Jurewicz et al. [57], this dose most likely causes a minimal toxic effect on fish tissues and, at the same time, has a strong chelating ability to lead nitrate in water.

Concerning lead nitrate LC_50_ values recorded in this study, calculations were performed for each fish species across the four different groups, as illustrated in Table 1. The mortality percentage of tilapia fish and catfish increased with increasing lead concentrations. The 96-h LC_50_ for Nile tilapia and African catfish was determined to be 5 mg/l, consistent with the LC_50_ values reported by Kim et al. [58]. This explains that heavy metal toxicity depends on the exposure route, duration, and the absorbed dose [59–61]. 

Lead is a biologically non-essential metal for living organisms. However, continuous lead exposure causes various physiological, behavioral, and biochemical alterations in fish [62]. Most clinical manifestations were observed in the tilapia fish group exposed to lead. This is related to the higher sensitivity of *Oreochromis* species than *Clarids* [63]. The observed clinical signs align with those of Azua and Akaahan [64], who observed erratic swimming behavior upon fish exposure to lead nitrate due to its effect on brain cells, causing several neurological changes [65, 66].

Additionally, the presence of melanosis in the lead-exposed fish group reflects the stress experienced by the fish during the experimental duration. This stress can elevate the levels of catecholamines and corticosteroids, leading to physiological skin darkening [67]. Similarly, findings from Nwobi et al. [68] indicate that lead exposure can reduce various essential bone minerals, resulting in growth retardation and skeletal deformities, as shown in Fig. 4. In contrast, no abnormal clinical signs were observed in the Si-MNPs and Pb + Si-MNPs groups, consistent with the observations made by Karlsson et al. [69].

The liver plays a crucial role in lead accumulation and detoxification of xenobiotics, and any disruption in its normal function can lead to fish mortality [70, 71]. Liver enzymes are stress indicators for confirming diagnoses and evaluating tissue damage caused by environmental pollution and metal intoxication [72]. Lead exposure has been directly linked to elevated levels of ALP and GGT [73]. Our findings revealed a significant increase in ALP and GGT levels in the serum of Nile tilapia and African catfish in the Pb groups compared to other experimental groups (Table 2).

This increase in ALP and GGT levels has been reported in various fish species following lead intoxication, such as *Oreochromis niloticus* [48, 74], *Cyprinus carpio* [72, 75], African catfish [63], Mystus fish species [76], and *Lethrinus harak* fish [77]. Simultaneous elevation of ALP and GGT levels typically occurs in hepatobiliary diseases, cholestasis, hepatocellular necrosis, and hepatic dysfunction resulting from long-term lead exposure [78–80]. ALP elevation is commonly associated with biliary obstruction, while elevated GGT confirms the hepatogenic origin of increased ALP [81]. In some cases, ALP elevation is concurrently associated with liver and bone disorders, which could explain the observed skeletal deformities in some tilapia fish in the lead-intoxicated group [82].

Long-term exposure of fish to heavy metals can lead to kidney dysfunction [83, 84]. Changes in urea and creatinine values are considered indicators of the adverse impact of lead on kidney function mechanisms. Lead is absorbed directly from the gills into the bloodstream, distributed throughout the body tissues, and primarily excreted through the kidneys [85]. This direct absorption has an immediate effect on the glomerular filtration rate, as it leads to the production of reactive oxygen species, ultimately causing kidney dysfunction and nephrotoxicity [86].

Our study found an increase in urea levels in Pb-exposed tilapia and catfish groups (Table 2), consistent with findings by El-Khadragy et al. [87] and El-Khayat et al. [88]. Creatinine levels showed a significant increase only in tilapia fish serum, likely due to severe glomerular damage caused by lead exposure [63, 89]. In contrast, no significant elevation was recorded in creatinine levels measured in catfish serum after 3 weeks of exposure to lead nitrate, possibly due to the more resistant and hardy nature of *Clarias* species to aquatic pollutants than Nile tilapia [64]. Elevated urea and creatinine levels due to lead toxicity have been reported in several fish species, such as *Cyprinus carpio* and *Oreochromis niloticus* [75, 90].

Our results indicated improved liver and kidney function parameters in the Si-MNPs and Pb + Si-MNPs groups. This suggests that stabilized silica and magnetite nanoparticles protect against lead-induced hepato-renal damage [49].

Aquatic organisms readily absorb lead and are subsequently involved in the bioaccumulation process through the food chain [2, 91]. This leads to oxidative stress, primarily caused by the production of free radicals, which causes numerous disorders and excessive damage [92, 93]. Oxidative stress is an imbalance between the production of reactive oxygen species (ROS) and the cell’s ability to detoxify reactive intermediates and repair damage that may occur in cellular molecules, increasing ROS production and decreasing defense mechanisms [25, 94]. Although ROS is typically produced by the cell, oxidative stress can be caused by various external factors, such as exposure to heavy metals [95].

Changes in the activity of antioxidant enzymes and the accumulation of oxidative damage products serve as crucial markers for oxidative stress. Reduced levels of GSH and other thiols render cells more susceptible to oxidative damage, while heightened activity of antioxidant enzymes can partially mitigate this effect [96].

Our results support these findings, showing a significant decrease in GSH concentration in the Pb group’s liver, gills, and muscles compared to the control group in both tilapia and catfish, as shown in Table 3. GSH is a scavenging non-enzymatic antioxidant compound that, when present in low concentrations due to rapid utilization in the presence of high levels of ROS, makes fish more susceptible to oxidative damage [97]. The removal of H_2_O_2_ is a critical defense strategy of aquatic organisms against oxidative stress [98]. In our study, the concentration of GSH significantly increased in the Pb + Si-MNPs group compared to the control (Table 3).

These findings align with Alfakheri et al. [99], who observed a considerably lower GSH concentration in the Pb group compared to the control group. Similarly, Loveline et al. [100] found that lead increased oxidative stress in *C. gariepinus* compared to the control group. Additionally, Saliu and Bawa-Allah [101] noted a decrease in GSH, SOD, and CAT in African catfish (*C. gariepinus*) when exposed to Pb (NO_3_)_2_. Furthermore, Olagoke [102] reported lower levels of GSH and GST in exposed fish compared to the control group.

The production of MDA serves as a marker of lipid peroxidation resulting from the breakdown of polyunsaturated fatty acids due to oxidative stress [103]. Our data demonstrated a significant increase in the concentration of MDA in the liver, gills, and muscles of the Pb group compared to the control group in both tilapia and catfish. Conversely, its concentration significantly improved, nearly reaching the control value, in the Pb + Si-MNPs group (Table 4).

The liver plays a significant role in the accumulation and detoxification of heavy metals, which may be linked to the high concentration of these metals in the liver [104]. Our findings align with the higher MDA levels observed in catfish exposed to lead toxicity, as reported by Maiti et al. [105].

Protein carbonyl is generally associated with oxidative stress-induced protein damage, as indicated by various diseases or tissue lesions [106]. Therefore, PC can be a marker for enzyme breakdown, amino acid structure modifications, and protein function alterations [107]. Our results in Table 5 demonstrate the impact of lead on protein oxidation, with the concentration of PCC significantly increasing in the liver, gills, and muscles of the Pb group compared to the control group in both tilapia and catfish. However, its concentration significantly improved, nearly reaching the control value in the Pb + Si-MNPs group. These findings suggest that metal pollutants may induce protein damage due to oxidative stress.

Similar results were reported by Neeratanaphan et al. [108], who found higher levels of PC in catfish from a landfill reservoir. Additionally, Ibrahim [109] showed that all fish tissues exposed to HgCl_2_ had significantly elevated carbonyl protein levels.

Reactive oxygen species are produced due to reactions accelerated by heavy metals. These reactions can damage tissues and macromolecules such as DNA, proteins, and lipids through oxidative stress. Using DNA fragmentation as a monitoring technique for heavy metal pollution was suggested by Moussa et al. [110]. Our data showed a significant increase in the percentage of DNA fragmentation in the Pb group in the liver, gills, and muscles compared to the control group. However, this percentage significantly decreased in all tissues in the Pb + Si-MNPs group compared to the Pb group (Table 6).

These findings align with Sultana et al. [111], who established a link between DNA fragmentation and heavy metal levels in various fish tissues. Moreover, Mohamed et al. [112] noted that tilapia exposed to higher heavy metal levels in severely polluted areas showed a higher frequency of DNA fragmentation in the gills, liver, and muscles, potentially indicating a lack of effective DNA repair systems. Furthermore, studies by Moussa et al. [110], Jindal and Verma [113], and Ratn et al. [114] have shown that fish exposed to toxins for prolonged periods exhibit increased DNA damage.

Numerous studies indicated that silica nanoparticles (Si-NPs) contribute positively to plant growth and development, particularly under stressful conditions. Silica has found application in environmental remediation for eliminating metals, non-metals, and radioactive elements, filtering water, and minimizing the discharge of brine, heavy metals, and radioactive substances into water bodies. Research has shown that silica nanoparticles can mitigate oxidative stress by stimulating the excess production or expression of non-enzymatic antioxidant metabolites and enhancing the functions of antioxidants [115].

In this study, the Pb + Si-MNPs demonstrated significantly higher GSH concentrations, lower MDA and PCC levels, and reduced DNA fragmentation (Tables 3, 4, 5 and 6). Rajkumar and Tennyson [116] suggested that elevated GSH content can act as an initial defense mechanism against toxic heavy metals and may help avoid oxidative stress [117].

Lead accumulation was assessed in the liver, gills, and muscles of Nile tilapia and African catfish at the end of the experimental period. Our findings, illustrated in Fig. 5a, indicate that tilapia exhibited the highest lead accumulation in liver tissues, consistent with previous studies by Salman [45], Dural et al. [118], Souid et al. [119], and Zhai et al. [120] in various fish species. This heightened accumulation in tilapia liver tissue may be attributed to the liver’s natural ability to produce significant amounts of metallothionein, a low metal-binding protein crucial for heavy metal uptake and detoxification [121, 122].

Conversely, in catfish (Fig. 5b), elevated lead content was observed in the gills. Numerous studies have noted this trend, particularly following water-borne exposure in different fish species such as *Tilapia zillii* [123], *Clarias gariepinus* [104, 124], starry flounder (*Platichthys stellatus*) [125], *Solea vulgaris* [126], and common carp *Cyprinus carpio* [127]. The gills’ larger surface area, directly exposed to external water pollutants, facilitates rapid diffusion and absorption of toxic metals through respiration and osmoregulation mechanisms [124, 128]. Additionally, *Clarias gariepinus* possesses accessory respiratory organs (AROs) that may enhance the trapping, absorption, and accumulation of Pb content from water, reducing Pb distribution in liver and muscle tissues.

The variation in metal tissue storage and concentration among different fish species can be attributed to factors such as the pathway and rate of Pb uptake (water-borne or dietary exposure) and elimination, fish feeding habits (pelagic or benthic feeder), duration of exposure, fish age, size, and length, metabolic activity, and various water parameters including temperature, salinity, and other interacting agents [129].

A significant concern is directed to the public health implications of lead, its accumulation in fish meat, and its safety for human consumption [130, 131]. Our study indicated that lead accumulation in the muscle tissues of Nile tilapia and African catfish is notably low compared to other tissues, as noted by Victor et al. [124], Lee et al. [129], and El-Moselhy et al. [132]. Furthermore, the Pb + Si-MNPs group exhibited lower Pb residues in both tilapia and catfish muscles than the Pb-exposed group. This reduction may be attributed to the potent chelating ability of silica-stabilized magnetite (Si-M) nanoparticles in binding Pb ions [133].

The histopathological changes observed in the tissues of tilapia and catfish exposed to lead (Figs. 6, 7 and 8, and 9) align with significant alterations in liver and kidney function, oxidative stress parameters, and observed genotoxicity [22, 77, 134–136]. Previous research by Patnaik et al. [137] has described neurotoxic histopathological effects on fish brains due to lead exposure, including vacuolation and gliosis, linked to Pb-induced oxidative damage, glycolysis, and mitochondrial dysfunction.

The proliferative tissue response seen in the gills of the lead-exposed group corresponds with findings from studies by Parashar and Banerjee [138] and Muñoz et al. [139], indicating a direct local effect of Pb on gills. Similarly, hepatocellular vacuolation and necrosis, indicative of lead toxicity in fish, have been previously reported by Suiçmez et al. [140] and Khidr et al. [141], likely due to energy depletion and reduced protein synthesis [142]. Kidneys, crucial for detoxification, are targeted by heavy metals. Muñoz et al. [139] noted histopathological changes in the kidneys similar to those in our study.

The observed increase in melano-macrophage centers in the spleen of the Pb-exposed group may be attributed to their role in detoxification and involvement in innate and adaptive immunity [143]. Conversely, the Pb + Si-MNPs groups exhibited minimal tissue pathology, possibly due to the antioxidant properties of iron nanoparticles countering lead ion damage [144], along with the high magnetic affinity of silica-stabilized magnetite (Si-M) nanoparticles for lead in water, reducing fish absorption [145].

## Conclusions

In conclusion, silica-stabilized magnetite (Si-M) nanoparticles exhibit a strong capability to chelate lead nitrate in water, thereby reducing lead absorption by fish. This property helps mitigate lead’s detrimental effects on hepatorenal function, oxidative stress parameters, genotoxicity, and histopathological changes. Additionally, it minimizes lead accumulation in fish muscle, ultimately enhancing fish health and performance and supporting sustainable aquaculture practices without negatively impacting human health.

## Data Availability

The data that support the findings of this study are available from the corresponding author upon request.
